# TrmFO, a Fibronectin-Binding Adhesin of *Mycoplasma bovis*

**DOI:** 10.3390/ijms18081732

**Published:** 2017-08-09

**Authors:** Yongpeng Guo, Hongmei Zhu, Jiayao Wang, Jing Huang, Farhan Anwar Khan, Jingjing Zhang, Aizhen Guo, Xi Chen

**Affiliations:** 1 College of Veterinary Medicine, Huazhong Agricultural University, Wuhan 430070, China; gyp18771951786@gmail.com (Y.G.); hongmeizhu429@gmail.com (H.Z.); hzauwjy@gmail.com (J.W.); jinghuang@webmail.hzau.edu.cn (J.H.); farhan82@aup.edu.pk (F.A.K.); jjzhang@webmail.hzau.edu.cn (J.Z.); aizhen@mail.hzau.edu.cn (A.G.); 2 The State Key Laboratory of Agricultural Microbiology, Huazhong Agricultural University, Wuhan 430070, China; 3 Hubei International Scientific and Technological Cooperation Base of Veterinary Epidemiology, Huazhong Agricultural University, Wuhan 430070, China

**Keywords:** *Mycoplasma bovis*, TrmFO, fibronectin, adhesion

## Abstract

*Mycoplasma bovis* is an important pathogenic mycoplasma, causing the cattle industry serious economic losses. Adhesion is a crucial step in the mycoplasmas’ infection and colonization process; fibronectin (Fn), an extracellular matrix glycoprotein, is a molecular bridge between the bacterial adhesins and host cell receptors. The present study was designed to characterize the Fn-binding ability of methylenetetrahydrofolate-tRNA-(uracil-5-)-methyltransferase (TrmFO) and its role in *M. bovis* cytoadherence. The *trmFO* (*MBOV_RS00785*) gene was cloned and expressed in *E. coli* BL21, and polyclonal antibodies against the recombinant TrmFO (rTrmFO) were raised in rabbits. Immunoblotting demonstrated that TrmFO was an immunogenic component, and the TrmFO expression was conserved in different *M. bovis* isolates. The mycoplasmacidal assay further showed that in the presence of complement, rabbit anti-recombinant TrmFO serum exhibited remarkable mycoplasmacidal efficacy. TrmFO was detected in both the *M. bovis* membrane and cytoplasm. By ligand dot blot and enzyme-linked immunosorbent assay (ELISA) binding assay, we found that rTrmFO bound Fn in a dose-dependent manner. Immunostaining visualized by confocal laser scanning microscopy showed that rTrmFO had capacity to adhere to the embryonic bovine lung (EBL) cells. In addition, the adhesion of *M. bovis* and rTrmFO to EBL cells could be inhibited by anti-rTrmFO antibodies. To the best of our knowledge, this is the first report to characterize the Fn-binding ability of TrmFO and its role in the bacterial adhesion to host cells.

## 1. Introduction

*Mycoplasma bovis* is the causative agent of bovine mycoplasmosis which results in pneumonia, mastitis, arthritis and genital disorders [1,2]. *M. bovis* was first isolated from the milk of cattle with mastitis by Hale in 1961 in the USA [3], and this pathogen has resulted in enormous economic losses to the cattle industry worldwide. In 2008, during an outbreak of *M. bovis* pneumonia and arthritis in China, *M. bovis* strain Hubei-1 and HB0801 were isolated, and the complete genome of both strains has been sequenced [4,5]. Due to the lack of genetic tools and other advanced research techniques, knowledge concerning the pathogenesis of *M. bovis* is relatively scarce, seriously hampering development of potential therapeutic and prophylactic measures [3].

Mycoplasmas evolved from Gram-positive bacteria through a process of regressive evolution, resulting in limitation of their biosynthesis capacities [6]. Most mycoplasmas display strict host and tissue specificities [7]. Adherence of mycoplasmas to host cells is a critical step in the initial phase of infection and an absolute requirement for successful colonization. Adhesins serve as vital virulence attributes in mycoplasmas, and decline of adhesion ability generally attenuates the bacteria [8]. Several adhesion proteins have been identified in *M. bovis*, including a 32 kDa membrane-localized protein P26 [9], a plasminogen binding enzyme α-enolase [10], NADH oxidase [11] and several variable surface lipoproteins (VspA, VspB, VspE, VspF and VpmaX) [12,13]. Identification and characterization of other adhesins will facilitate a better understanding of interactions between *M. bovis* and host cells.

Fibronectin (Fn), a multifunctional extracellular matrix glycoprotein, exists in soluble and matrix forms in diverse body fluids and tissues, and the binding of Fn is associated with bacterial colonization, bacteria-host interactions and virulence behavior [14,15]. It is suggested that Fn-binding proteins (FnBPs) can promote bacterial adhesion to host cells via a sandwich model in which Fn acts as a molecular bridge between surface exposed FnBPs and integrins on host cells [14,16]. Several FnBPs have been identified in mycoplasmas, including elongation factor Tu (EF-Tu), glyceraldehyde-3-phosphate dehydrogenase (GAPDH) and pyruvate dehydrogenase A-C (PDH A-C) in *Mycoplasma pneumoniae* [17,18], Mhp182 and Mhp271 in *Mycoplasma hyopneumoniae* [19,20], enolase in *Mycoplasma synoviae* [21], PlpA and Hlp3 in *Mycoplasma gallisepticum* [22], and cytoadhesive lipoprotein T (LppT) in *Mycoplasma conjunctivae* [23]. These findings highlight the possibility that there may also be FnBPs in *M. bovis*, which might contribute to *M. bovis* infection in and persistence on host cells.

Recently, our laboratory developed an effective attenuated *M. bovis* vaccine through passage of *M. bovis* HB0801 150 times in vitro [24]. Although the underlying mechanisms of attenuation of the *M. bovis*-150 strain (CCTCC No.: M2011102) remain unclear, it can be speculated that the down-regulation and the absence of some virulence factors could be responsible for the attenuation. Methylenetetrahydrofolate-tRNA-(uracil-5-)-methyltransferase (TrmFO) is a conserved flavin dinucleotide (FAD) binding protein, which has been identified as the member of tRNA modification enzymes responsible for the folate-dependent m^5^U-54 biosynthesis [25]. Using an iTRAQ-based quantitative proteomic analysis, we found that expression of TrmFO was down-regulated in the attenuated *M. bovis*-150 strain compared to the virulent strain *M. bovis* HB0801 (unpublished data). Among these down-regulated proteins in the attenuated *M. bovis*-150 strain, NADH oxidase and variable lipoprotein VspX have been reported to contribute to *M. bovis* adhesion to host cells [11,13]. In the light of this, the aim of current study was to characterize the moonlight function of TrmFO in the pathogenesis of *M. bovis*.

## 2. Results

### 2.1. Bioinformatics Analysis

In *M. bovis* HB0801, the 1284-bp ORF of *trmFO* (*MBOV_RS00785*) gene encoded a 427-amino-acid protein with a molecular weight of 48.8 kDa and isoelectric point of 6.43. *M. bovis* HB0801 TrmFO shared more than 98% amino acid level identity with TrmFO from other sequenced *M. bovis* strains, while it exhibited ≤80% homology with TrmFO from other mycoplasmal species (Table 1). TrmFO contained no conventional signal peptide or transmembrane domain. TrmFO was predicted to be a cytoplasmic protein by subcellular localization predictor (CELLO).

### 2.2. Expression and Purification of Recombinant TrmFO (rTrmFO)

**Figure 1:**
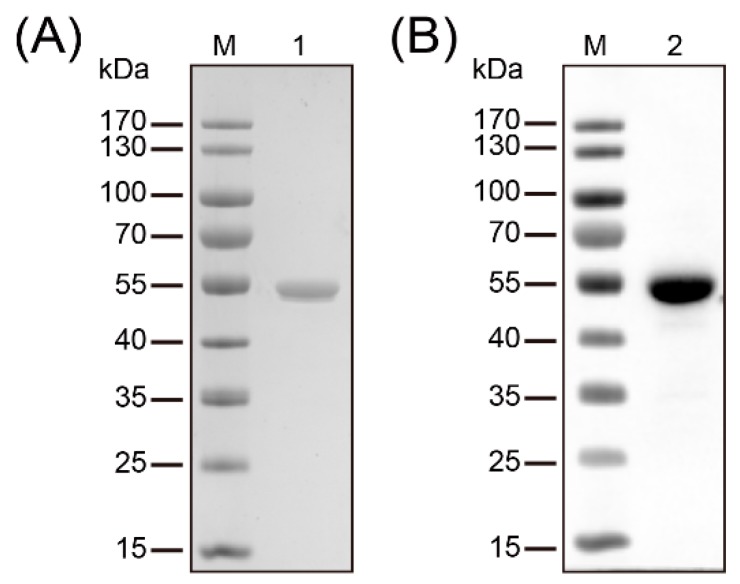
Purification and identification of recombinant methylenetetrahydrofolate-tRNA- (uracil-5-)-methyltransferase (TrmFO). (**A**) SDS-PAGE analysis confirmed the purity of the recombinant TrmFO (rTrmFO). M: Molecular weight marker; Lane 1: Purified rTrmFO. (**B**) Immunoblotting analysis of the rTrmFO using mouse anti-His tag antibodies. M: Molecular weight marker; Lane 2: rTrmFO.

The full-length *trmFO* was amplified from *M. bovis* HB0801 and cloned into plasmid pET-30a (+) to facilitate the expression and purification of recombinant TrmFO (rTrmFO). After induction by 0.8 mM IPTG, the overexpression of rTrmFO in *E. coli* BL21 resulted in a protein with apparent molecular size of 54 kDa (Figure 1A). The observed increase in the molecular size of rTrmFO over the native TrmFO was attributed to its N-terminal His-tag. Purified rTrmFO was recognized by mouse anti-His tag antibody on immunoblotting analysis (Figure 1B).

### 2.3. Immunogenicity Analysis of rTrmFO

**Figure 2:**
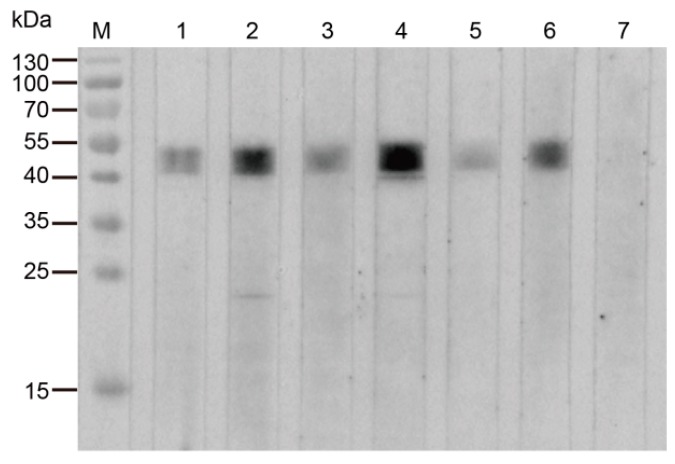
Immunoblotting analysis of the purified rTrmFO with individual *M. bovis*-positive and *M. bovis*-negative sera. **M**: Molecular weight marker; **Lanes 1** to **3**: Individual positive sera from experimentally *M. bovis* infected cattle; **Lanes 4** to **6**: Individual positive sera from naturally *M. bovis*-infected cattle; **Lane 7**: *M. bovis*-negative serum.

*M. bovis*-positive sera recognized the rTrmFO, whereas *M. bovis*-negative serum did not display reactivity with the recombinant protein on western blotting (Figure 2). The results suggested that *M. bovis*-infected cattle generated specific antibodies against TrmFO, thereby indicating that the TrmFO was an immunogenic component in *M. bovis*.

### 2.4. Expression of TrmFO in Different M. bovis Isolates

**Figure 3:**
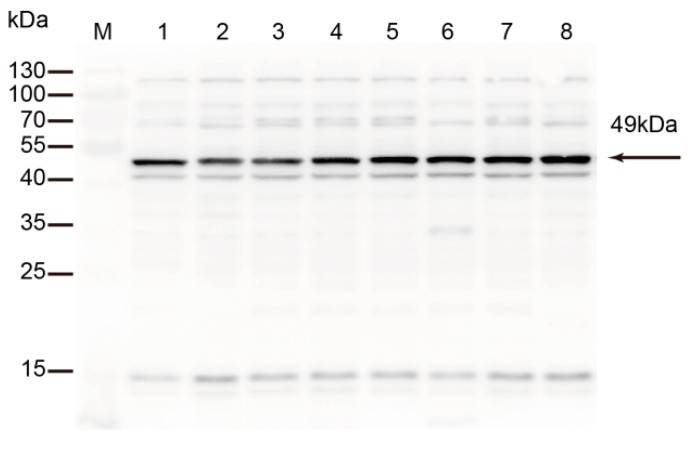
Detection of TrmFO in different strains of *M. bovis*. Western blotting analysis was conducted with 20 μg of whole cell proteins in each lane. **M**: Molecular weight marker; **Lanes 1** to **8**: *M. bovis* strain HB0801, PG45, XM, JXXY, BZ, NNH, WX and YC, respectively.

Eight *M. bovis* strains originating from different districts and lesions were analyzed. Rabbit anti-rTrmFO polyclonal antibodies strongly reacted with a 49 kDa protein corresponding to TrmFO in all tested *M. bovis* strains (Figure 3).

### 2.5. Mycoplasmacidal Activity of Rabbit Anti-rTrmFO Serum

**Figure 4:**
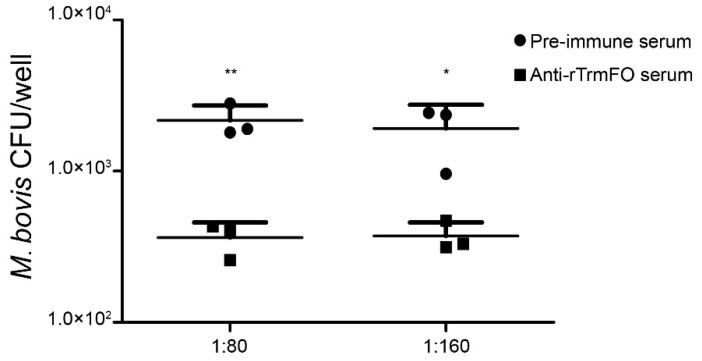
Mycoplasmacidal activity of rabbit anti-rTrmFO serum. *M. bovis* HB0801 was incubated with rabbit pre-immune or anti-rTrmFO serum (diluted 1:80 and 1:160) in the presence of complement. Data represent the mean ± SEM from three separate experiments. * and ** represent *p* < 0.05 and *p* < 0.01, respectively.

Rabbit anti-rTrmFO serum exhibited strong mycoplasmacidal activity mediated by a complement system. Compared to the rabbit pre-immune serum, incubation of the anti-rTrmFO serum with *M. bovis* HB0801 in the presence of the complement resulted in effective killing of the bacteria (Figure 4).

### 2.6. Subcellular Localization of TrmFO in M. bovis

**Figure 5:**
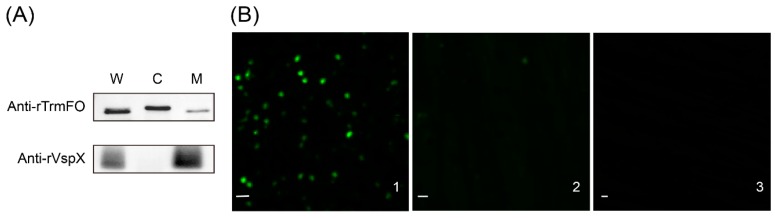
Subcellular localization of TrmFO in *M. bovis*. (**A**) Western blotting analysis. W: whole cell proteins; C: cytoplasmic proteins; M: membrane proteins. (**B**) Immunofluorescence assay. **B1**: *M. bovis* cells were incubated with rabbit anti-rTrmFO serum. **B2**: *M. bovis* cells were incubated with rabbit pre-immune serum. **B3**: *M. bovis* cells were incubated with PBS. Bars, 2 μm.

TrmFO was detected in the membrane, cytoplasm, and whole cell proteins of *M. bovis* (Figure 5A), indicating that TrmFO was localized in both *M. bovis* cell membrane and cytoplasm. The control monoclonal antibodies against rVspX only reacted with the membrane and whole cell proteins, confirming that the membrane and cytoplasm protein fractions were free of cross contamination.

In the immunofluorescence assay (IFA) with rabbit anti-rTrmFO antibodies, staining of *M. bovis* with fluorescein isothiocyanate was observed (Figure 5B1), whereas IFA with pre-immune serum exhibited little staining (Figure 5B2) and with PBS no signal (Figure 5B3), suggesting that TrmFO was membrane-localized.

### 2.7. Fibronectin (Fn)-Binding Ability of rTrmFO

**Figure 6:**
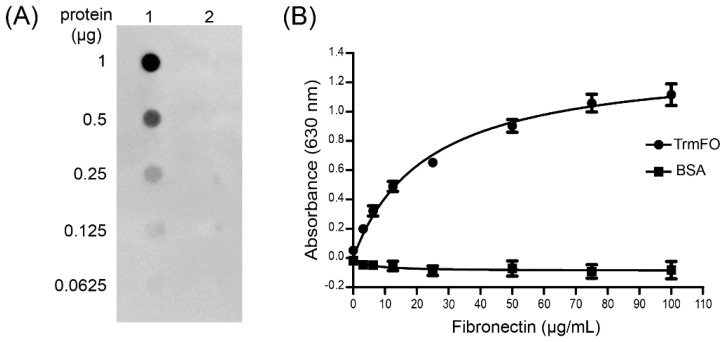
Characterization of the fibronectin (Fn)-binding ability of rTrmFO. (**A**) Ligand dot blot assay demonstrated the Fn-binding ability of rTrmFO. **1**: rTrmFO; **2**: bovine serum albumin (BSA). (**B**) Binding of Fn to immobilized rTrmFO in microplate. All data represent the mean ± SEM of triplicate readings from three independent experiments (*n* = 9).

To determine whether *M. bovis* TrmFO was a Fn binding protein, ligand dot blot and an enzyme-linked immunosorbent assay (ELISA) binding assay were performed. In the ligand dot blot assay, TrmFO exhibited Fn-binding activity (Figure 6A, lane 1), while no interaction was observed between Fn and bovine serum albumin (BSA) under similar conditions (Figure 6A, lane 2). It was further confirmed by ELISA that immobilized rTrmFO bound to Fn in a dose-dependent manner (Figure 6B).

### 2.8. Direct Adhesion of rTrmFO to EBL Cells and Adhesion Inhibition

**Figure 7:**
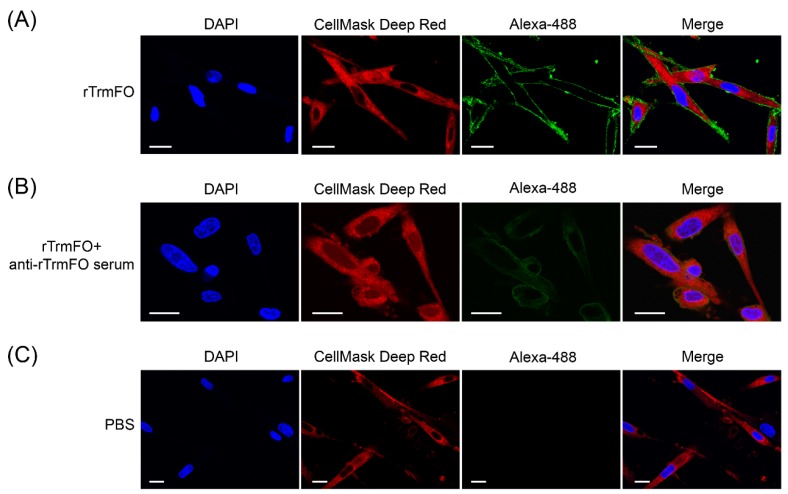
Confocal laser scanning microscopy depicting rTrmFO adhesion to EBL cells and adhesion inhibition of rabbit anti-rTrmFO serum. EBL cells were fixed, incubated with rTrmFO and immunostained with rabbit anti-rTrmFO polyclonal antibodies and donkey anti-rabbit IgG (whole molecule)-Alexa 488. Cell nuclei and membranes were labeled with 4′,6-diamidino-2-phenylindole (DAPI) and CellMask Deep Red, respectively. (**A**) The rTrmFO exhibited adhesion ability to EBL cells. (**B**) The rTrmFO was pre-incubated with anti-rTrmFO serum, then this mixture was added to the fixed cells. The adherence process was significantly inhibited by rabbit anti-rTrmFO antibodies. (**C**) PBS was used as a negative control. Bars, 20 μm.

**Figure 8:**
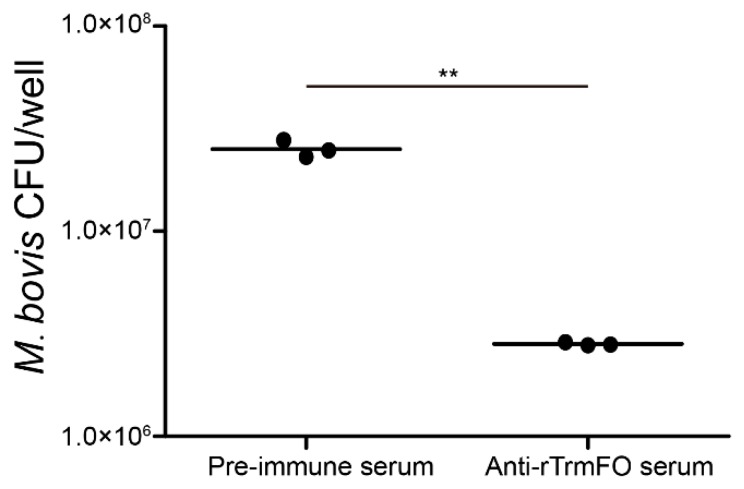
The adhesion of *M. bovis* to EBL cells was inhibited by rabbit anti-rTrmFO serum. *M. bovis* was incubated with rabbit pre-immune or anti-rTrmFO serum (diluted 1:50 in PBS). Data represent the mean ± SEM from three separate experiments. ** represents *p* < 0.01.

The adhesion of rTrmFO to EBL cells was determined by laser scanning confocal microscopy. rTrmFO was shown to be capable of adhering to the fixed EBL cells (Figure 7A). After pre-incubation with rabbit anti-rTrmFO serum, rTrmFO adherence to EBL cells was significantly inhibited (Figure 7B). The PBS control showed no adherence to EBL cells (Figure 7C). *M. bovis* adhesion to EBL cells could be significantly inhibited by rabbit anti-rTrmFO serum (Figure 8).

## 3. Discussion

By means of 150 passages of the wild-type *M. bovis* strain HB0801, we recently created an avirulent *M. bovis*-150, which was confirmed to exhibit protective ability against *M. bovis*-related pneumonia in calves [24]. TrmFO, a FAD-binding protein responsible for catalyzing the site-specific formation of 5-methyluridine in position 54 (m^5^U54) of tRNA in bacteria, has been identified to be down-regulated in the attenuated *M. bovis*-150 strain. Homology analysis indicated that TrmFO was identical at amino acid level among sequenced *M. bovis* strains and other mycoplasmas. Additionally, the recombinant TrmFO could be specifically recognized by sera of naturally and experimentally *M. bovis*-infected cattle. The antigenic conservation of TrmFO was also confirmed by immunoblotting, in which rabbit anti-rTrmFO antibodies strongly reacted with a 49 kDa protein corresponding to TrmFO in all *M. bovis* isolates tested. In the mycoplasmacidal assay, we found that rabbit anti-rTrmFO serum displayed effective *M. bovis* killing capacity in the presence of complement. Currently, great efforts have been made in exploiting novel immunodominant antigens of *M. bovis*, and several important antigenic proteins have been found including variable surface proteins (Vsp), lipoprotein P48, Hsp60, MbovP579, lipase A (MilA), PDHB, and GAPDH [12,26,27,28,29,30,31]. Among these, MbovP579, PDHB and MilA have been applied as serological diagnostic markers of *M. bovis* infection. Meanwhile, a subunit vaccine based on GAPDH has been shown to induce high IgG1 titers but failed to protect cattle exposed to *M. bovis* [32]. The results of the current study suggested that TrmFO, as a highly conserved antigen within the *M. bovis* cluster, could be a promising candidate for developing effective DNA or protein subunit vaccines. However, further studies in vivo are needed to determine whether TrmFO can elicit a protective immune response against *M. bovis* infection.

Neither transmembrane domains for surface anchoring nor classical secretory signal peptides were found on *M. bovis* TrmFO. However, by western blotting, TrmFO was detected in both the cell membrane and cytoplasm. The surface localization of *M. bovis* TrmFO was further confirmed by immunofluorescence assay. The dual cellular distribution of TrmFO suggested that the protein may carry out a moonlighting function in *M. bovis*. A growing number of cytoplasmic proteins have been shown to actively participate in bacterial adhesion and colonization processes when expressed on the cell surface [33]. GAPDH and enolase are two widely studied bacterial glycolytic enzymes with adhesion capacity. GAPDH has been shown to play an important role in bacterial selective adhesion to host components, including epithelial cells, extracellular matrix proteins, cytoskeleton proteins, and so on [34]. Enolase has been characterized as a plasminogen receptor in *Mycobacterium tuberculosis* [35], *Streptococcus pneumoniae* [36], *Bacillus anthracis* [37], and pathogenic mycoplasmas [10,38,39,40], facilitating the proteolytic plasmin activity of these pathogens, which potentially promotes their tissue invasion and dissemination in hosts. Apart from glycolysis enzymes, there are also proteins involved in other metabolic pathways that have been found to be translocated to cell membrane and contribute to bacterial virulence. NADH oxidase is an adhesion-related factor in *Streptococcus pneumoniae*, and immunization of mice with recombinant NADH oxidase has been shown to induce a protective immune response against *S. pneumoniae* [41]. In *M. pneumoniae,* EF-Tu was surface-exposed and displayed fibronectin binding ability [17]. Similar to these moonlighting proteins, we have provided evidence in the current study that TrmFO is a binding partner of bovine fibronectin and serves as an adhesin in *M. bovis*.

In the absence of a rigid cell wall, the pathogenicity of mycoplasmas is highly dependent on the cytoplasmic membrane which is in direct contact with host cells during attachment [42]. Fibronectin is a glycoprotein widely present in the extracellular matrix and body fluids, which contain binding sites for various extracellular molecules, including fibrin, gelatin, heparin and adhesion receptors [14]. In this context, capture of fibronectin via surface-anchored adhesins is a common strategy for bacteria to strengthen their adhesion to and invasion of host cells [16]. In terms of mycoplasma species, dose-dependent Fn recognition was first observed in *M. penetrans* [43], followed by *M. pneumoniae*, in which the carboxyl region of EF-Tu interacted with Fn [44]. *M. conjunctivae* LppT was shown to contain an RGD (Arg-Gly-Asp) motif that is a specific binding site for both Fn and beta heparins of eukaryotic host cells [23]. Using ligand dot blot and ELISA binding assay, we demonstrated that *M. bovis* TrmFO possessed fibronectin binding ability. Work is currently in progress to identify amino acid sequence motifs that correspond to the fibronectin-binding property of TrmFO. More importantly, TrmFO was found to be a novel adhesin in *M. bovis*, based on the direct adhesion and inhibition assay in which rTrmFO was observed to adhere to EBL cells and the adhesion was inhibited by anti-rTrmFO polyclonal antibodies. Furthermore, the adhesion of *M. bovis* to EBL cells could also be reduced when the bacteria were pre-incubated with anti-rTrmFO serum. Comparative genomics of *M. bovis* strains reveals a 14.2-kb deleted region covering 14 genes in the attenuated *M. bovis*-150 strain [45]. However, the production of H_2_O_2_, a significant virulence related factor in *mycoplasma* species, was not affected by the mutations specific to the genes in the 14.2-kb deleted region. In fact, the down-regulation of virulence factors like TrmFO should be partly responsible for the attenuation of the *M. bovis*-150 strain. As far as we know, this is the first report to characterize the fibronectin binding property of bacterial TrmFO. In addition, we have shown that the protein exerts an additional function on *M. bovis* adhesion to EBL cells. The findings suggest that TrmFO is of great importance in the *M. bovis*-host cell interaction. The knowledge of the interplay among *M. bovis* adhesins, fibronectin and host cell receptors will provide a further piece in the puzzle of bacterial invasion and persistence in different bovine cell types.

## 4. Materials and Methods

### 4.1. Ethics Statement

The animal experiment protocols in this study were in strict accordance with the Hubei Regulations for the Administration of Affairs Concerning Experimental Animals. Animal experiments were approved by the Hubei Province Science and Technology Department, which is responsible for experimental animal ethics. All experiments were supervised by the Scientific Ethical Committee for Experimental Animals of Huazhong Agricultural University, Wuhan, China (Permit Number: HZAURAB-2015-006).

### 4.2. Bioinformatics Analysis

Amino acid identity matches were conducted with BLASTP [46]. SignalP 4.1 Server was used to predict the presence of signal peptide [47]. Prediction of transmembrane helices in TrmFO was performed with TMHMM Server V. 2.0 [48]. CELLO was employed to predict protein subcellular localization [49].

### 4.3. Bacterial Strains, Cultivation Conditions and Cell Line

*M. bovis* strain HB0801 was isolated from a calf lung with pneumonia and stored at the China Center for Type Culture Collection (CCTCC; M2010040). The strain was cultivated in an atmosphere of 37 °C, 5% CO_2_ on a pleuropneumonia-like organism (PPLO) agar plate or in PPLO broth containing 2.1% (*w*/*v*) PPLO broth, 0.5% (*w*/*v*) yeast extract, 0.1% (*w*/*v*) sodium pyruvate, 0.001% (*w*/*v*) phenol red, 20% donor equine serum (Hyclone, South Logan, UT, USA), and 400,000 IU/L penicillin-G.

*Escherichia coli* strains DH5α and BL21 (TransGen Biotech, Beijing, China) were cultured at 37 °C on Luria-Bertani (LB) agar plates or in LB broth supplemented with 30 μg kanamycin/mL as selection needed. *E. coli* DH5α was used as the host cell for DNA manipulation, while *E. coli* BL21 was used for the expression of recombinant protein. The embryonic bovine lung (EBL) cells, certified to be free of mycoplasma contamination, were cultured in MEM supplemented with 15% fetal bovine serum (Gibco, Sydney, Australia), 100 IU penicillin/mL, and 100 μg streptomycin/mL.

### 4.4. Cloning, Expression and Purification of rTrmFO

*M. bovis* HB0801 chromosomal DNA was used as template for amplification of the *trmFO* gene. TGA is a universal termination codon but it encodes tryptophan in mycoplasmas. When cloning a mycoplasmal gene in the *E. coli* expression system, the presence of TGA codon can lead to the early termination of gene translation. In this study, the TGA codon is not found in the coding sequence of the *trmFO* gene. Specific primers for amplifying *trmFO* were designed with Oligo 7 software (Molecular Biology Insights, West Cascade, CO, USA) as follows: F5′-GGCGGTACCATGAAAAAAATAAGAGTTATTG-3′ and R5′-CGGGGATCCCTATAAATTTTGCTTAATAAAC-3′ (underlined sequences represent *Kpn*I and *BamH*I restriction sites, respectively). The PCR product was cloned into pET-30a (+) vector (Novagen, Madison, WI, USA). His-tagged TrmFO was expressed in *E. coli* BL21 and purified by Ni-NTA columns affinity chromatography under native conditions according to the manufacturer’s instructions (GE Healthcare, Boston, MA, USA). The purified rTrmFO was analyzed by SDS-PAGE and by western blotting using mouse anti-His tag antibodies (Cell Signaling Technology, Danvers, MA, USA). Protein concentration was quantified using a BCA protein assay kit (Thermo Fisher Scientific, Waltham, MA, USA).

### 4.5. Preparation of Polyclonal Antibodies Against rTrmFO

Antiserum to rTrmFO was raised in New Zealand White rabbits by subcutaneous immunization with 1 mg of purified rTrmFO emulsified in Freund’s complete adjuvant (only for the first immunization) or Freund’s incomplete adjuvant (Sigma, St Louis, MO, USA). The immunization was conducted three times at 2-week intervals. The antibody titers were measured by ELISA. Briefly, the 96-well plates were coated with purified rTrmFO (100 ng/well) at 4 °C overnight. After being washed three times with PBST, unoccupied sites were blocked with 5% skim milk in PBST for 1 h at 37 °C. The two-fold serial dilution (from 1:1 × 100 to 1:2^22^ × 100) of rabbit serum anti-rTrmFO and pre-immune serum were added to wells. Then, bound antibodies were detected by incubation with horseradish peroxidase (HRP)-labeled goat anti-rabbit IgG (diluted 1:4000) for 1 h. Rabbits were sacrificed via cardiac bleeding to collect positive antiserum 10 days after the third immunization. The antibody titer to rTrmFO was 1:2^16^ × 100 (positive serum OD: negative serum OD ≥ 2.1). The polyclonal antibodies against rTrmFO were purified using a HiTrap Protein G affinity column (GE Healthcare, Boston, MA, USA).

### 4.6. Immunoblotting Analysis

Immunoblotting was performed to analyze whether TrmFO played an immunogenic role using *M. bovis* positive sera. Purified rTrmFO was electrophoretically separated on a 12% SDS-PAGE gel and then transferred onto the PVDF membrane (Millipore Corp, Billerica, MA, USA). The membrane was blocked with 5% skim milk in TBS at 4 °C overnight. The blots were washed with TBST (1 mL/L Tween-20, 100 mM Tris-Cl, 9 g/L NaCl, pH 7.5) and incubated with bovine sera (diluted 1:100 in TBST, 6 positive and 1 negative *M. bovis* sera) at room temperature for 1 h. Later, bound antibodies were detected by incubation for 1 h with horseradish peroxidase (HRP)-labeled goat anti-bovine IgG (SouthernBiotech, Birmingham, AL, USA) (diluted 1:4000 in TBST). Color development was performed using Chemiluminescent Substrate (Thermo, Fisher Scientific, Waltham, MA, USA). Then the signals were detected on the Image DNR MF-ChemiBIS (NDR, Jerusalem, Israel).

To assess the expression of TrmFO in different *M. bovis* isolates, whole cell proteins of eight *M. bovis* strains (Table S1) were transferred to the PVDF membrane and probed with rabbit anti-rTrmFO polyclonal antibodies (diluted 1:2000). Subsequently, the membrane was washed and incubated with HRP-labeled goat anti-rabbit IgG (diluted 1:4000). Immunoreactive bands were developed using Chemiluminescent Substrate.

### 4.7. Complement Dependent Mycoplasmacidal Assay

Rabbit pre-immune serum and anti-rTrmFO serum were inactivated at 56 °C for 30 min and used to test for bactericidal activity against *M. bovis* HB0801 by a complement-mediated bactericidal assay as previously described with some modifications [22]. Briefly, 50 μL of rabbit pre-immune serum or antiserum was added to the wells of a 96-well plate; then, 30 μL of bacterial suspension (~10^5^ colony-forming unit/mL) in Dulbecco’s PBS containing calcium, magnesium, and 0.1% gelatin (DPBSG) and 20 μL of diluted complement (1:10 in DPBSG) were added. Following incubation at 37 °C for 1 h, the mixture was tenfold serially diluted in PPLO medium and plated onto PPLO agar plates. The plates were incubated at 37 °C for 48 h with 5% CO_2_, and colonies were counted under a microscope. Three independent experiments were conducted in triplicate.

### 4.8. Subcellular Localization of M. bovis TrmFO

To identify the subcellular distribution of *M. bovis* TrmFO, membrane and cytoplasmic proteins of *M. bovis* HB0801 were extracted by glycerol-osmotic lysis with a slight modification [50]. Briefly, 1 L of mycoplasmas in mid-log phase was harvested by centrifugation at 12,000× *g* for 15 min. Cell pellets were washed with 0.25 M NaCl three times prior to resuspension in 5 mL of 2 M glycerol. Cell suspensions were then lysed by rapid transference into 50 volumes of preheated, deionized water and incubated at 37 °C for 15 min with shaking. Membrane fractions were collected by centrifugation at 60,000× *g* for 45 min, and sequentially washed in deionized water, followed by 0.25 M NaCl, and again deionized water. Membranes were further purified by centrifugation through 30–60% continuous sucrose gradient at 160,000× *g* for 4 h at 4 °C and collected by a syringe. Cytoplasmic fractions were concentrated by passing an ultrafiltration column (Millipore Corp, Billerica, MA, USA). Proteins were quantified with a BCA Protein Assay Kit. Twenty micrograms of total, membrane and cytoplasmic proteins were separated by 12% SDS-PAGE and transferred onto PVDF membranes. After being washed three times with TBST, unoccupied sites were blocked with 5% skim milk in TBST for 1 h at 37 °C, followed by incubation with rabbit anti-rTrmFO polyclonal antibodies (diluted 1:500 in TBST) at 4 °C overnight. HRP-labeled goat anti-rabbit IgG were used as secondary antibodies (SouthernBiotech, Birmingham, AL, USA) (diluted 1:4000 in TBST). To test possible cross-contamination between the membrane and cytoplasmic proteins, monoclonal antibodies against VspX (membrane protein, self-made) (diluted 1:2000 in TBST) were used [13].

For the immunofluorescence assay, *M. bovis* was cultured in PPLO broth medium to log-phase and washed three times with PBS. The intact *M. bovis* cells (~10^10^ CFU) were incubated at 37 °C for 1.5 h with rabbit anti-rTrmFO serum (1:100 dilution in PBS), pre-immune serum (1:100) or PBS. Cells were washed with PBS three times, and the samples were incubated with donkey anti-rabbit IgG (whole molecule)-Alexa 488 (AntGene, Wuhan, Hubei, China) diluted 1:300 in PBS at 37 °C for 1 h. Following five washes with PBS, the cells were dispersed in 300 μL of PBS and visualized under a fluorescence microscope (ZEISS, Oberkochen, Germany).

### 4.9. Fibronectin-Binding Assays

Ligand dot blot and ELISA binding assay were conducted to characterize the binding ability of rTrmFO to bovine Fn. A dot blot binding assay using a Bio-Dot microfiltration apparatus (Bio-Rad, Hercules, CA, USA) was performed as previously described by Jenkins et al. with some modifications [51]. Purified rTrmFO or BSA was twofold serially diluted from a concentration of 10 μg/mL to 0.625 μg/mL. Following this, 100 μL of proteins were spotted onto a nitrocellulose membrane (Millipore Corp, Billerica, MA, USA). The membrane was removed from the microfiltration apparatus and washed with TBST three times prior to being blocked with 5% skim milk in TBST at room temperature for 4 h. After being washed three times in TBST, the blot was exposed to 10 μg bovine Fn/mL (Millipore Corp, Billerica, MA, USA) diluted in 1% skim milk-TBST at 4 °C overnight. After washing, the membrane was incubated with rabbit anti-bovine Fn IgG (Millipore Corp, Temecula, CA, USA) at a dilution of 1:3000 in 1% skim milk-TBST for 1 h, followed with goat anti-rabbit IgG-HRP (diluted 1:4000 in TBST). The membrane was incubated with Chemiluminescent Substrate, then the signals were detected on the Image DNR MF-ChemiBIS.

For the ELISA binding assay, 96-well plates were coated with purified rTrmFO (500 ng/well) in carbonate coating buffer (18 mM NaHCO_3_, 27 mM Na_2_CO_3_, pH 9.6). After incubation at 4 °C overnight, unoccupied sites were blocked with 5% skim milk in PBST for 1 h at 37 °C. After being washed five times with PBST, wells were incubated with varying concentrations of bovine Fn (1.562, 3.125, 6.25, 12.5, 25, 50, 75 and 100 μg/mL in 1% skim milk-PBS) at 37 °C for 1.5 h. Bound Fn was detected by the addition of rabbit anti-bovine Fn IgG diluted 1:500 in 1% skim milk-PBS, followed by goat anti-rabbit IgG-HRP (diluted 1:6000). Wells incubated with BSA were used as negative control for Fn binding. The bound HRP was detected with 3,3,5,5-tetramethylbenzidine (TMB) solution for 10 min and stopped with 2 M H_2_SO_4_. Absorbance was measured at 630 nm with a microplate reader. Nonspecific adherence observed in wells without proteins coated was subtracted from all readings. Graphpad Prism version 5 was used to construct graphs.

### 4.10. Adherence and Inhibition Assays

Direct adhesion of rTrmFO to EBL cells was visualized by confocal laser scanning microscopy (CLSM) as described by Zou et al. with some modifications [13]. Briefly, the EBL cells were cultured for 24–36 h at 37 °C. After washing, EBL cells were fixed with 4% paraformaldehyde (PFA) at room temperature (RT) for 15 min and then blocked with 1% donor equine serum in PBS at RT for 1 h. Subsequently, the cells were washed five times with PBST and incubated in 1 mL of PBS containing 90 μg of rTrmFO for 1 h at 4 °C. For the adherence inhibition assay, the rTrmFO was pre-incubated with 20 μL of rabbit anti-rTrmFO serum in 1 mL of PBS at 4 °C for 1 h prior to adding it to the fixed cells. The cells incubated with 1 mL of PBS without rTrmFO were used as a negative control. The rTrmFO was used as soon as possible after purification to ensure its biological activity. After excessive washing to remove non-adherent protein, the bound protein was stained with rabbit anti-rTrmFO polyclonal antibodies diluted 1:100 in 1% equine serum PBS for 1 h, followed by donkey anti-rabbit IgG (whole molecule)-Alexa 488 diluted 1:300 for 1 h. Immunofluorescence was detected using a Carl Zeiss LSM 510 CLSM.

The inhibition of *M. bovis* adhesion to EBL cells by rabbit anti-rTrmFO serum was performed as previously described with some modifications [38]. Briefly, EBL cells were inoculated into 24-well plates (1.25 × 10^5^ cells/well) and grown to a confluent monolayer. After washing, EBL cells were blocked with 1% BSA-MEM at 37 °C for 30 min. Subsequently, EBL cells were infected with *M. bovis*, which had been pre-incubated with rabbit pre-immune serum or anti-rTrmFO serum for 2 h at 4 °C, at a multiplicity of infection (MOI) of 1000 for 30 min at 37 °C. Following four washes with PBS to remove unbound *M. bovis*, EBL cells were liberated with 0.25% Trypsin in MEM. The bound *M. bovis* were tenfold serially diluted and plated on PPLO agar plates for counting bacterial colonies. Three independent experiments were performed in triplicate.

### 4.11. Statistical Analysis

Data are expressed as the mean ± SEM of at least three independent experiments, and the statistical analyses were performed using Student’s *t*-test. Significant differences were denoted as * *p* < 0.05 and ** *p* < 0.01.

## Figures and Tables

**Table 1:** Amino acid sequence identity of *M. bovis* HB0801 methylenetetrahydrofolate-tRNA- (uracil-5-)-methyltransferase (TrmFO) with those of other mycoplasmas.

Species	Identity	NCBI Protein ID
*Mycoplasma bovis* Hubei-1	100%	AEI89864.1
*Mycoplasma bovis* CQ-W70	100%	AIA33742.1
*Mycoplasma bovis* PG45	98%	ADR24753.1
*Mycoplasma agalactiae* PG2	80%	CAL58845.1
*Mycoplasma putrefaciens* KS1	56%	AEM68895.1
*Mycoplasma* sp. HU0214	55%	KNG79513.1
*Mycoplasma yeastsii* GM274B	54%	AJM71909.1
*Mycoplasma capricolum* subsp. *capricolum* 14232	48%	KEZ18460.1
*Mycoplasma mycoides* subsp. *capri str.* GM12	48%	ACU78480.1

NCBI, the National Center for Biotechnology Information.

## Supplementary Materials

Supplementary materials can be found at www.mdpi.com/1422-0067/18/8/1732/s1.
