# Supplementary material for: TrmFO, a Fibronectin-Binding Adhesin of Mycoplasma bovis
Source: Int J Mol Sci. 2017 Aug 9;18(8):1732. doi: 10.3390/ijms18081732 (PMC5578122; doi:10.3390/ijms18081732)
Supplement: Supplementary file 1 [file ijms-18-01732-s001.pdf]

Supplementary Table S1. Strains of *M. bovis* tested for the expression of TrmFO

Strain	Country of origin	Date of isolation	Lesions
HB0801	China	2008	Lung
PG45	USA	1961	Udder
XM	China	2009	Lung
JXXY	China	2012	Lung
BZ-NHD0982	China	2008	Lung
NNH-NHD0956	China	2010	Throat
WX-NHD0964	China	2013	Synovial fluid
YC-NHD0967	China	2013	Udder
